# Electroceuticals for peripheral nerve regeneration

**DOI:** 10.1088/1758-5090/ac8baa

**Published:** 2022-09-08

**Authors:** Woo-Youl Maeng, Wan-Ling Tseng, Song Li, Jahyun Koo, Yuan-Yu Hsueh

**Affiliations:** 1 School of Biomedical Engineering, Korea University, Seoul 02841, Republic of Korea; 2 Division of Plastic and Reconstructive Surgery, Department of Surgery, National Cheng Kung University Hospital, College of Medicine, National Cheng Kung University, Tainan 701, Taiwan; 3 Division of Plastic and Reconstructive Surgery, Department of Surgery, Tainan Hospital, Ministry of Health and Welfare, Tainan 700, Taiwan; 4 Department of Bioengineering and Medicine, University of California, Los Angeles, Los Angeles, CA 90095, United States of America; 5 Interdisciplinary Program in Precision Public Health, Korea University, Seoul 02841, Republic of Korea; 6 International Research Center for Wound Repair and Regeneration, National Cheng Kung University, Tainan 701, Taiwan; 7 Center of Cell Therapy, National Cheng Kung University Hospital, College of Medicine, National Cheng Kung University, Tainan 701, Taiwan

**Keywords:** peripheral nerve regeneration, electroceuticals, electrical stimulation, nerve conduit, conductive conduit

## Abstract

Electroceuticals provide promising opportunities for peripheral nerve regeneration, in terms of modulating the extensive endogenous tissue repair mechanisms between neural cell body, axons and target muscles. However, great challenges remain to deliver effective and controllable electroceuticals via bioelectronic implantable device. In this review, the modern fabrication methods of bioelectronic conduit for bridging critical nerve gaps after nerve injury are summarized, with regard to conductive materials and core manufacturing process. In addition, to deliver versatile electrical stimulation, the integration of implantable bioelectronic device is discussed, including wireless energy harvesters, actuators and sensors. Moreover, a comprehensive insight of beneficial mechanisms is presented, including up-to-date *in vitro, in vivo* and clinical evidence. By integrating conductive biomaterials, 3D engineering manufacturing process and bioelectronic platform to deliver versatile electroceuticals, the modern biofabrication enables comprehensive biomimetic therapies for neural tissue engineering and regeneration in the new era.

## 1. Introduction

**Figure 1.**
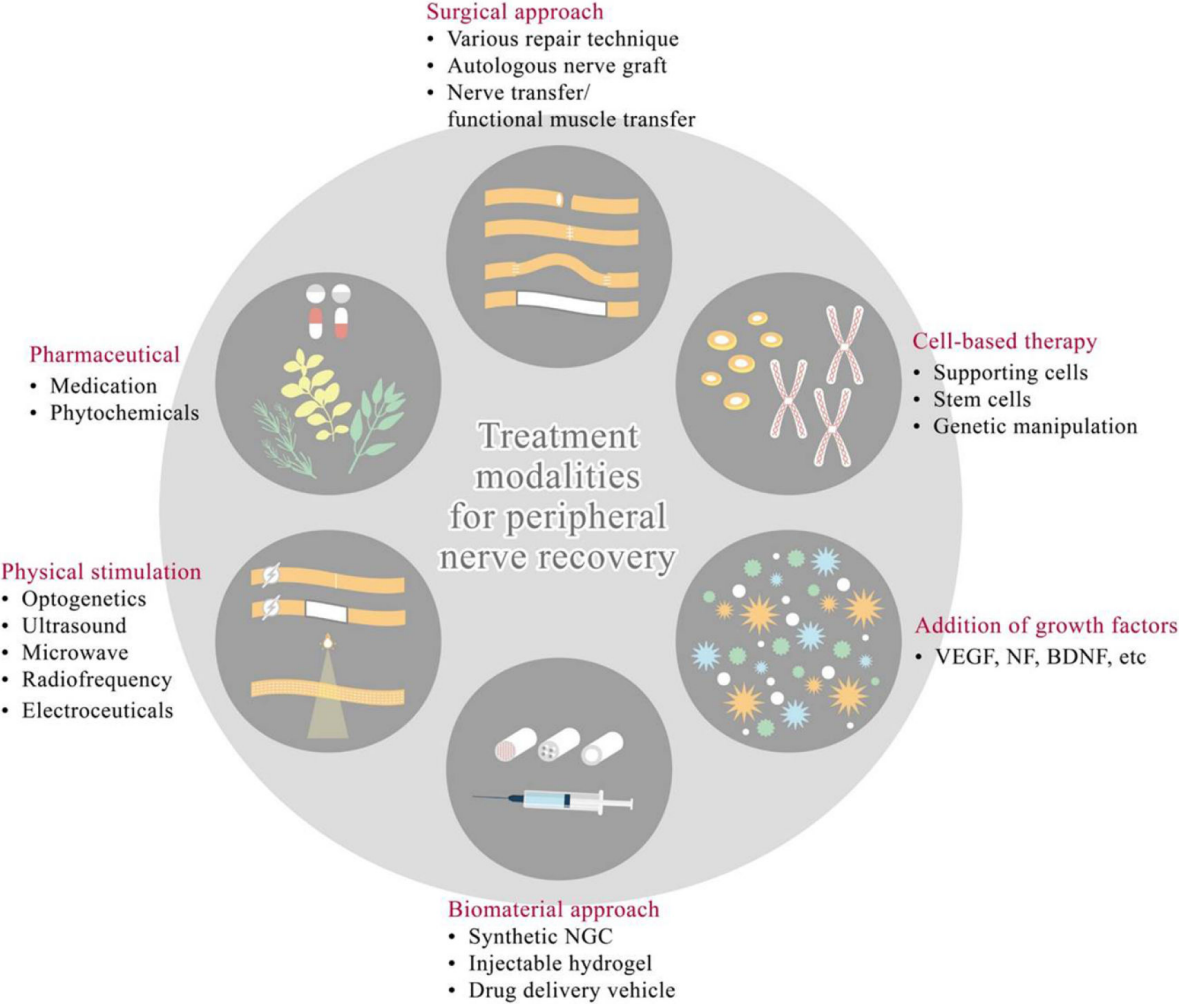
Current treatment options for promoting PNR. The state-of-art of multi-modality approaches to repair or reconstruct PNI include surgical, non-surgical and physical stimulation. Surgical intervention includes all kinds of microsurgical repair, nerve graft, nerve/muscle transfer. Non-surgical approach includes pharmaceuticals, various synthetic growth factors and cell-based therapies. Physical stimulation consists of optogenetics, ultrasound, microwave, radiofrequency and electroceuticals. Biomaterial approaches include synthetic NGCs, hydrogel and controlled release drug-containing vehicles.

Peripheral nerve injuries (PNIs) constitute 2%–5% of trauma cases, leading to significant economical and psychosocial burden to the individuals and society [1-3]. For severe injuries, PNIs adversely affect behavior, mobility, sensations, perception of skin, muscle and joints resulting in long-term disability. Unlike central nervous system (CNS) injuries, which commonly yield failure of injured axons to regenerate, PNIs are followed by robust regeneration with higher possibility of recovery of sensory and motor functions [4]. The frequency of axon regeneration is dependent on various factors including alterations within the cell body, stability of growth cone, and the hindrance of damaged tissue between neuron and its end organ. In humans, axonal regeneration is typically shown as 1–2 mm d^−1^ without additional treatments that can effectively accelerate the process [5]. This regenerative response is associated with widespread transcriptional and epigenetic changes in injured neurons [6, 7]. If the regeneration fails, however, the end organ such as motor unit would undergo irreversible degeneration 12–18 months after denervation [8]. Treatments of PNIs consist of surgical and non-surgical approaches and numerous modalities have been developed since the unsatisfactory outcome of severe PNIs remains a noteworthy clinical issue [9, 10] (figure 1). Among non-surgical modalities, numerous works have been studied including medication and phytochemicals [1, 11, 12]. Surgical therapeutic approaches for peripheral nerve recovery comprise a variety of techniques including direct repair [13, 14], nerve grafting (autografts, allografts) [15, 16], nerve transfer [17, 18], fibrin glue [1, 14, 19], nerve conduits [14, 20-30] and cell-based therapy [12, 31-33]. Advantages and disadvantages of each technique have been widely discussed [1, 10]. Currently, novel treatments that have been reported include phytochemicals, optogenetics, fat grafting, tissue-engineered nerve grafting and electrical nerve stimulation [6, 34-37].

For PNIs with a nerve defect or a gap needed to be bridged, autologous nerve graft remains the gold standard treatment. However, autografts have the drawback of donor site morbidity and limited supply. Other alternate methods are thus extensively explored. Nerve guidance conduits (NGCs) with the ease of various design such as combination of cell-based therapy or adjustment of the microenvironment with growth factors, gene therapy or tissue-engineered graft are increasingly being considered as an alternative to nerve autografts [22, 29, 31, 38].

Electroceuticals deliver electrical impulses targeting the neural circuits that regulate the body’s organs and functions, via either wearable or implantable electronic device [39, 40]. As a new category of novel therapeutic approach, electroceuticals have applied and demonstrated therapeutic potential for ischemic stroke [41], Alzheimer’s disease [42], type I diabetes [43], wound healing [44], cardiovascular regulation [45], gastrointestinal tract disorder [46] and even developmental disorders [47]. In the field of peripheral nerve regeneration (PNR), electrical stimulation (ES) as a therapeutic intervention for PNIs has been studied for decades. Percutaneous ES has long been clinically applied as prevention of muscular atrophy [48]. As for ES focused on injured nerve after repair, the positive effects of brief, low-frequency ES on PNR was established in various animal experiments [12, 35, 49]. This review will provide comprehensive information on (a) current animal and human evidence of ES therapy; (b) fabrication of conductive materials and electronic devices; and (c) integration of these two fundamental components of electroceutical approaches.

## 2. Electroceuticals as novel approaches for PNR

The main pathologies of PNIs include a cascade of changes in both the local injured axon and the associated neurons. Wallerian degeneration, which initiates within 24–48 h following nerve injury, consists of active axon degeneration and myelin degradation at nerve distal to the injury site [50]. This process leads to the degradation of neurofilaments, the rearrangement of nerve’s cytoskeleton with detachment of axon terminals from the target. In the meantime, the neurons proximal to the injured sites undergo a form of polarized growth in order to reinnervate towards their targets [51, 52]. However, the injured neuron might undergo programmed cell death activated within 6 h in unfavorable situations regulated by various extrinsic and intrinsic factors [53].

This regenerative response is associated with wide-spread transcriptional and epigenetic regulations in the injured neurons, axons and target organs. Substantial advances have been established in terms of the coordinated actions of transcription factors, epigenetic modifiers and microRNAs, which are widely investigated in the peripheral nervous system in recent studies [4, 6, 7, 54]. To facilitate the extensive regenerative process after PNIs, a comprehensive strategy should be considered for systemwide neuromodulations from proximal neurons, local injured axons to distal target organs.

### 2.1. Beneficial mechanisms of ES for PNR

ES as an advanced therapeutic approach for PNIs, has been approved for its promising role in promoting PNR with specific target effect. Percutaneous ES has long been clinically applied for the prevention of muscular atrophy [48]. With direct stimulation on the proximal site to the injured nerve, brief low-frequency (20 Hz) ES has revealed beneficial effects in various animal experiments [12, 35, 49]. Gordon presented the first randomized controlled trial, demonstrating the beneficial therapeutic outcomes of proximal ES on patients with severe carpal tunnel syndrome after surgical decompression. The clinical evidence of direct nerve ES enables the additional proximal benefits on neuronal cell, in terms of promoting PNR during nerve surgery on injury site [35]. In contrast to low-frequency (20 Hz) ES, kilohertz stimulation lead to reversible nerve block under the same stimulation amplitude, which was usually being applied to alleviate neuropathic pain (see full review in [55, 56]).

**Figure 2.**
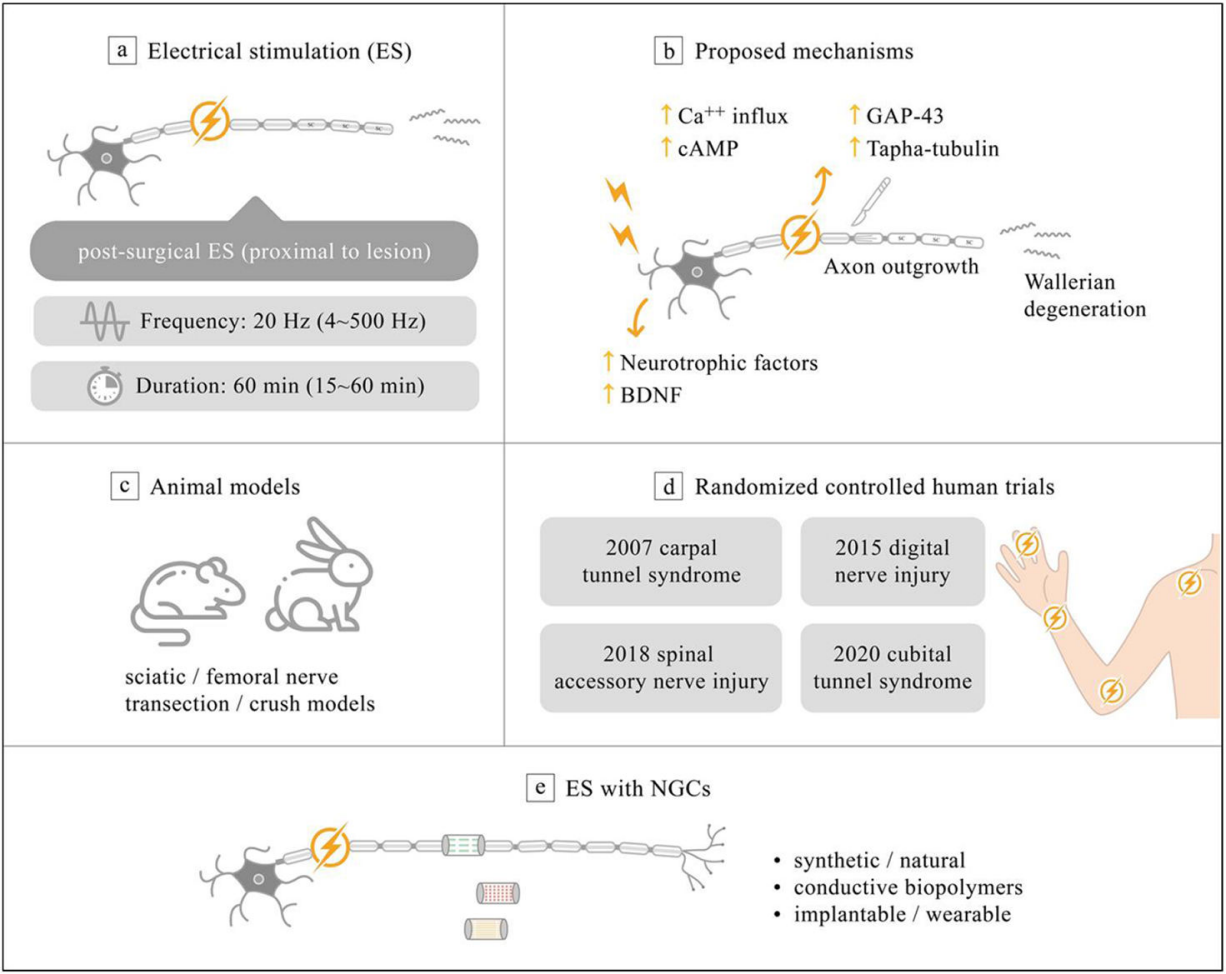
Current evidence of electroceuticals for PNR. (a) Therapeutic stimulation parameters and application of brief ES. (b) Proposed mechanism of peripheral nerve ES. (c) Current established *in vivo* evidence for transection/crush injuries at sciatic/femoral nerves of rodent and rabbits. (d) Current human evidence among median nerve [64], digital nerve [34], spinal accessory nerve [75] and ulnar nerve [76]. (e) Choice of NGC with electroceuticals.

ES has been widely demonstrated to offer benefits in the regeneration of bone, cartilage, skin, spinal nerves, and peripheral nerves [57]. The current evidence indicates postsurgical or intraoperative single proximal ES (SP-ES) as a promising therapeutic strategy to promote PNR after a variety of injuries [35, 49, 58-62]. SP-ES in which the electrical stimulus is applied directly on the nerve stump proximal to the injured site, is validated in various animal studies. A single dose of brief (1 h), low frequency (20 Hz) ES, has been proved to amplify axon regeneration after nerve transection and microsurgical repair (figure 2(a)) [35, 60, 63, 64]. Moreover, a recent research reveals that brief (10 min) SP-ES can provide identical therapeutic benefits to the abovementioned 60 min protocol in an acute sciatic nerve transection/repair rat model and thus imply the translational potential for future clinical application [65]. The current established mechanism of SP-ES contributing to the therapeutic effect of PNR involves increase of neurotrophic factors and upregulation of their receptors on neuronal cells, including brain derived neurotrophic factor (BDNF) [63, 66]. Subsequent elevation of intracellular cyclic adenosine monophosphate (cAMP) level and related cAMP response element binding protein pathway [67-69] further enhance expression of regeneration-associated genes, such as Talpha-1 tubulin and growth associated protein 43 (GAP-43), resulting in axon regeneration [70-72]. One *in vitro* study has demonstrated that ES increases production of nerve growth factor (NGF) from Schwann cell (SC), leading to efficient axon remyelination [73]. In addition, a recent *in vitro* study discovers that ES triggering the p38 mitogen-activated protein kinase pathway in PC12 mutant cells, which plays an important role in promoting neurite outgrowth (figure 2(b)) [74].

### 2.2. Current evidence of SP-ES after nerve injury

Numerous animal studies and human trials present the therapeutic benefits of PNR from SP-ES (figure 2(c)) [6, 35, 59]. In 1983, Nix and Hopf first described the positive effect from direct ES on injured nerves and the affected muscles. Better twitch force, tetanic tension, and muscle action potential were observed 2 weeks after ES (4 Hz, daily) treatment in a rabbit nerve transection model [77]. Further animal studies showed that postsurgical 1 h 20 Hz ES directly on injured nerve yield earlier recovery of motor and sensory functions in acute crush injury [78], transected femoral nerve [79, 80] and sciatic nerve in rats models [81, 82]. Moreover, in delayed nerve repair model of rats, a single session of 1 h ES at 20 Hz immediately after delayed nerve repair significantly increased the numbers of motoneurons reinnervating toward chronically denervated muscle [83]. Moreover, a recent research proposes that repetitive distal ES on nerve gap injury model, demonstrating the therapeutic potential on preservation of neuromuscular junction and improvement of motor function [84]. The current animal researches demonstrated the therapeutic evidence of SP-ES, whether for the injury mechanism (crush or transection), nerve type (femoral or sciatic nerve), treatment timing (acute or chronic) and stimulation site (proximal or distal to injured nerve).

For human therapeutic evidence, to our knowledge, there are currently four randomized controlled trials regarding the therapeutic benefits of SP-ES for PNR (figure 2(d)). Two randomized controlled trial had conducted to examined the effect of SP-ES immediately after carpal tunnel [64] and cubital tunnel syndrome [76] release, demonstrating motor unit number estimates increased significantly by postoperative 1 and 3 years as compared to the control unstimulated group, respectively. Wong *et al* further revealed immediate effect of SP-ES after digital nerve repair with improved digit sensation and nearly full functional recoveries [34]. For patients undergoing oncologic neck dissection, intraoperative SP-ES (continuous 60 min, 20 Hz) to spinal accessory nerve contributed to significant improvement in electrophysiologic outcome and overall shoulder function at 12 months after surgery [75]. Despite all the above-mentioned beneficial evidences of SP-ES, several translational challenges remain in terms of optimization of stimulation dosage, feasibility in critical nerve gap injury, and the potential benefit of preconditioning ES [85]. In addition, an innovative bioelectronic platform that combines such bi-directional ES is also crucial in order to achieve PNR in the future.

### 2.3. ES with conductive materials

Despite the abovementioned convincing evidence of proximal ES on PNR, the therapeutic evidence of ES on critical nerve gap remains unexplored due to limitations of biomaterials. NGC provides tissue engineered biomimetic tubular structures to bridge critical nerve gap when encountered and has drawn significant attention in terms of nerve tissue repair and regeneration (figure 2(e)). They can be made of natural or synthetic biopolymers such as chitosan, gelatin, collagen, polylactide, poly(lactic-co-glycolic acid) (PLGA), or polycaprolactone (PCL), which are designed to offer supportive mechanical and/or biochemical cues [29, 86, 87]. Either conductive or non-conductive biomaterials can be used as the scaffold of NGC. However, several issues should be still considered such as non-biodegradability, possible long-term *in vivo* toxicity, and non-homogeneous distribution of the conductive particles in neural tissue [21, 25, 29, 57, 88]. Recent development on implantable conductive materials and structure can offer the opportunity of delivering wide-spread ES from the implantation site, with the additional advantage of minimal stimulus needed as compared to transcutaneous approach. The efficacy of the combination of ES with NGC has been explored in literature [20, 57, 62, 86, 89, 90]. Choices of different types of conductive biomaterials with minimum toxicity and development of an implantable or wearable electronic device with numerous designs of interface are both hot topics in the field of neural tissue engineering [57, 87, 88, 91].

To engineer a novel NGCs for electroceuticals, the electrical conductivity of the fabricated biomaterials provides a compelling solution to the current clinical difficulties [92]. To achieve this goal, the optimal dosage of ES and protocol are both important determining factors for the therapeutic effects of neuromodulation. It is reported that SP-ES effectively accelerates the regenerating motoneurons with a low frequency of 20 Hz for an hour a day [79, 83, 93]. However, a recent study applying single or two sessions of proximal ES on critical nerve gaps, observed that such ES can only promote sensorimotor recovery at the first session of ES when delivered at the time of reconstruction, with no benefit of a second delayed session of ES 4 weeks after the initial reconstruction [94]. Therefore, the exploration of the application of repetitive ES and the integration of conductive conduit with ES would be of great value. Various ES conditions such as electrical conductivity of the NGCs, charged voltage, current, and duration have been summarized in table 1. From this perspective, the standard protocol has not yet been established. Although not as efficient as autograft, ES plus conductive conduit indeed demonstrates significant therapeutic benefits. The following section will introduce both the *in vitro* and *in vivo* influence of the PNR from the combination of ES and functional electrically conductive conduit.

#### 2.3.1. *Beneficial evidence of neural cell response on conductive materials* in vitro

Conductive NGCs aims to reconnect nerve defects physically and communicate biophysical signals for facilitating neural tissue outgrowth. Although conductive substrates support cellular activity with or without ES [106, 113], it has been found that they can enhance axon outgrowth when applied in conjunction with ES [90, 99]. Recently, it has been widely studied the effect of cell stimulation and behavior on electrically conductive materials. When electrical stimuli are applied to the injured nerves, the neuronal cells are activated, resulting in cellular responses such as proliferation, migration, differentiation, neurite outgrowth, and remyelination, which extensively influence the peripheral nervous system [114].

**Figure 3.**
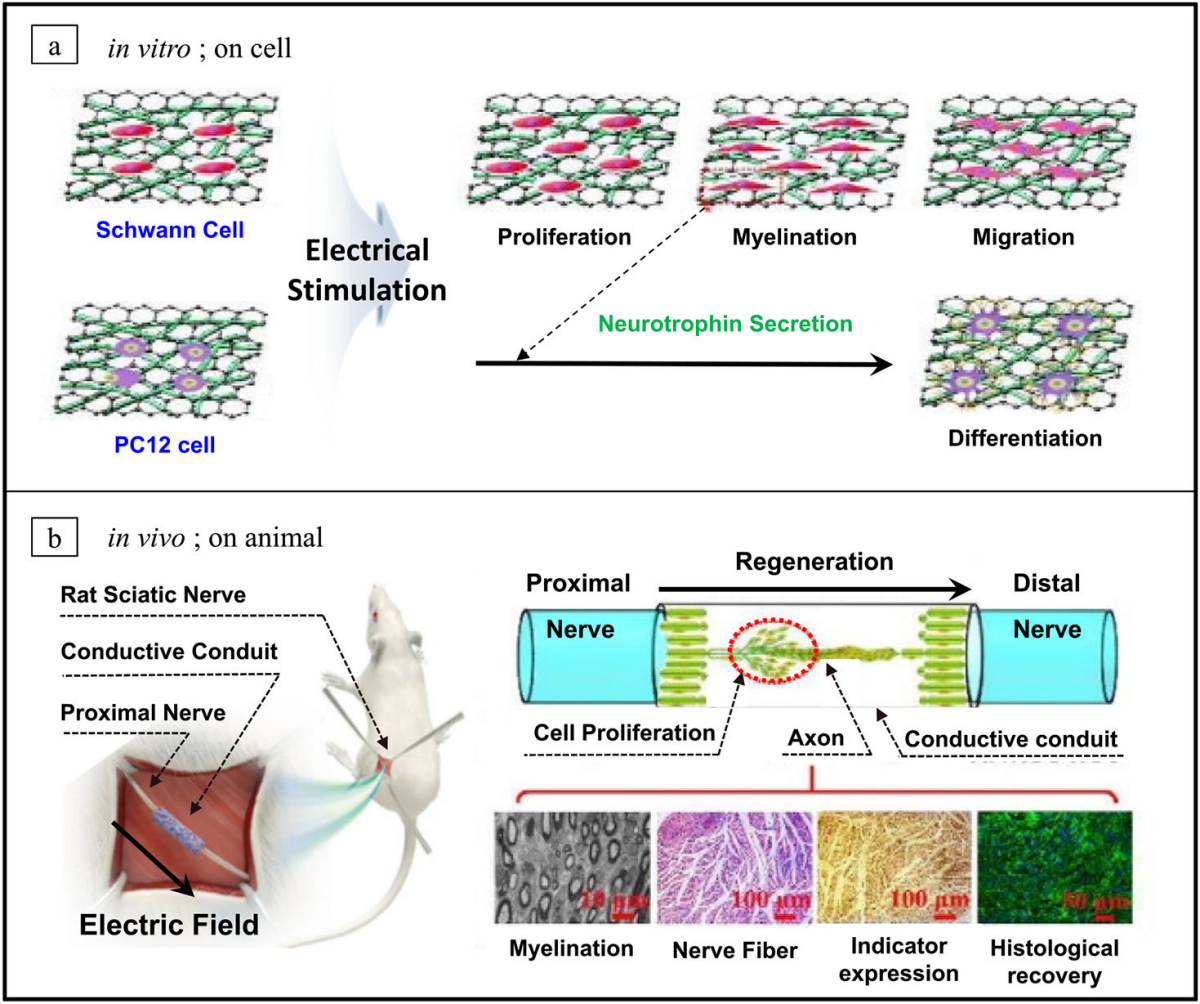
The *in vitro* and *in vivo* beneficial effects of neural regeneration with combined conductive conduit and ES. (a) Neural cell response on the electrical field; *in vitro.* Illustration of various cell activity by ES on the conductive substrate. Reprinted from [107], Copyright (2019) with permission from Elsevier. (b) Electroceuticals to accelerate nerve repair; *in vivo.* Illustration of phenomena by ES in sciatic nerve repair. From [112] Reprinted with permission from AAAS. Reprinted from [107], Copyright (2019) with permission from Elsevier.

SCs are a representative neuroglia cell to myelinate the axons in peripheral nervous system (PNS). It resides in peripheral nerve tissues, with important roles in chaperoning axon sprouting. More specifically, SCs activate proliferation and migration in the regeneration process by supporting axon growth and subsequent myelinization, resulting in nerve regeneration through the secretion of neurotrophic factors [115, 116]. Zhao *et al* evaluated the *in vitro* effect of ES on SCs, demonstrating enhanced viability, proliferation and migration, along with upregulated expression of neurotrophic factors (BDNF, NT-4/5, NGF, GDNF). Moreover, the constructed polypyrrole (PPy)/silk conductive NGC accompanying ES could effectively promote *in vivo* axonal regeneration and remyelination [98]. Accordingly, when NGCs is fabricated with electrical conductivity, it enables control of cell adhesion, migration and interaction of neural cells under an electric field. The *in vitro* cell responses in figure 3(a) showed that in the group adopting ES to the conductive matrix, SC growth, proliferation, and migration were significantly promoted than in the absence of ES. In addition, the remyelination by SCs was also facilitated by ES [107]. Figure 3(b) shows various histological benefits of conductive conduit with ES for sciatic nerve regeneration. It was observed that motor performance could be improved during rehabilitation by sciatic function index (SFI) analysis. This synergistic effect was more clearly demonstrated in the observation of immunostained images to evaluate regeneration of axonal growths in the conduits. With the electric field stimulation, the differentiation and elongation of neurons were enhanced on the conductive cross-linked poly(3,4-ethylene dioxythiophene) (PEDOT) substrate [117]. In addition, applied ES to the unidirectional aligned nanofiber matrix shows a synergetic effect for higher cell viability [98].

#### 2.3.2. *Electroceuticals to accelerate the nerve repair* in vivo

As summarized in table 1, many groups have demonstrated the effect of this strategy using the transected sciatic nerve model in Sprague–Dawley (SD) rats. Researchers have investigated the accelerated regeneration of the sciatic nerve using over 10 mm nerve gap models in 150–250 g adult rats. Conductive NGCs had shown considerable improvement compared to non-conductive NGC groups. ES used in non-conductive materials can only apply directly to cells and cannot provide a large area. Furthermore, the combinations of conductive NGCs with ES contributed to higher regenerative ability than the solely conductive NGCs group [89, 90, 95, 98, 107].

Song *et al* had demonstrated the beneficial effects of ES on 15 mm nerve gap injury bridged by conductive NGC (PPy/PLCL) [95]. It revealed remarkable regeneration capability, and there was identical outcomes in sciatic function index, nerve conduction velocity, distal compound motor action potential, and recovery rate of triceps muscle weight between the PPy/PLCL with ES group and autograft group. In addition, Huang *et al* explain that that localized ES enhances the migration ability of SCs that migrating into the conductive conduit (PPY/chitosan) paves the way for axon regeneration [90]. It has been shown that these ES therapies allow a synergistic effect not only through the electrical properties of the neural conduit but also through the longer and faster neural filaments with highly aligned micropatterns [98]. So, the remarkable potential of this integrated strategy as electroceuticals suggests a promising future direction in PNS regeneration.

In summary, the integrated strategy of conductive NGC with ES shows substantial regeneration capacity comparable to autograft transplantation. Electroceuticals with conductive materials are promising therapeutic options in the future, providing both material and electrophysiological cues to support consequential PNR.

## 3. Fabrication of bioelectronic conduit

Providing well-developed conductive materials into the injured site is a key biotechnology to have synergic efficacy of regeneration without side effects and secondary damages. Conductive conduit implanted between the transected nerves can serve to use as an electrically conductive pathway for bilateral stimulation in the proximal and distal. Therefore, a comprehensive understanding of conductive materials, optimal structure and fabrication will help us develop more effective electrotherapy. In this section, we discuss these issues for a promising therapeutic strategy and optimization.

### 3.1. Candidate raw materials for conductive nerve conduit

Nerve regeneration requires intrinsic ionized electric signals and is wrapped in a myelin sheath (nonconductor) to prevent leakage of ions from the axon to transmit biological signals. However, in severe nerve injuries, transplantation of synthetic scaffolds is required and axons are guided in specific directions. But so far, artificial structures have not yet been as conductivity natural ions and tissues. Therefore, for bioinspired-mimic properties, implantable conduits are recommended alternative candidate materials with electrical conductivity.

Conductive polymers are called *π*-conjugated polymers, resulting in a conductive biostructure due to mostly carbon bonding structures, forming a valence band and involving electrons moving easily [118]. These *π*-conjugated polymers, such as PPy, polyaniline (PANI), and PEDOT, exhibit conductivity when dopants are added through a redox reaction [119]. The electrical stimulus on the conductive polymers drives the dopant to move through the structure, creating polarons and allowing the charge to flow through [20]. However, these conductive polymers are not biodegradable and have poor solubility in most solvents. Currently, these conductive polymers are usually not used independently but used a combination with natural or synthetic polymers [89, 90, 95, 97, 98, 103, 120-124]. The biodegradable natural and/or synthetic polymers are partially absorbed, the non-degradable conductive polymers still retain the debris and circulate through the *in vivo* environment. Although the effect of short-term ES after surgery provides acceleration of nerve growth, it has limitation that is difficult to track non-degradable conductive polymer fragment after recovery [29]. For this reason, it could not be a complete alternative material despite the potential application of electrical signal transmission.

Carbon nanomaterial has high electrical conductivity and excellent mechanical properties. The nanosized structure and large surface area could serve as a promising strategy to enhance neuro-regeneration. Carbon nanotube (CNT) provide electrical conductivity in state of dispersing on nanofiber that lead to improving cell proliferation of PC12 and SCs [125, 126]. However, the biocompatibility of CNTs still remains argumentation due to poor clear evidence in safety. In several studies, authors do not agree that non-functionalized CNTs have biocompatibility to support neuronal growth and regeneration [113, 127, 128]. The studies of degradation rate, discharge, and safety of CNTs are remained works to overcome the current limitations in future applications. Likewise, graphene (GO) also improves the biological cue between the biocompatible scaffold and the cell membrane because GO has strong *π*-bonding and a large surface area, resulting in high electrical conductivity and promoting signal transduction and metabolic activity. Therefore, it significantly improves neuronal expression both *in vitro* and *in vivo.* Many studies support the remarkable regeneration effects by dispersing graphene oxide (GO) or reduced GO (rGO) in a scaffold matrix [106, 129, 130]. GO, when inserted into the body, tends to accumulate in organs such as the lungs, liver, and spleen, and exposure to GO can cause severe cytotoxicity and disease [131]. On the other hand, as the result that GO could be biodegraded *in vivo* by macrophages is still being presented [132], the debate about the application of carbon nanomaterials as biomaterials in the future is expected to continue.

Metal particles can be used as conductive biomaterials including nanoparticles of gold (Au), silver (Ag), and copper (Cu) [133, 134]. Composite materials, mixed with nano metal materials and hydrogel, improve mechanical strength and electrical conductivity, and the level of cell adhesion and proliferation are able to control by the concentration of metal material [135]. Furthermore, metals can completely and harmlessly dissolve, reabsorb, or degrade at the molecular level, known as transient electronics. Such transient metals include magnesium (Mg), zinc (Zn), tungsten (W), iron (Fe), molybdenum (Mo) [136]. These metals are considered good bioresorbable materials, but their corrosion mechanisms are completely different. There are concerns that consumption of oxygen and byproducts in the corrosion process may cause necrosis of the surrounding tissues, so a lot of care should be taken when employing metal materials [137]. Besides, the allowance of each metal is different in the organs, sex, and ages; therefore, quantitative and systematic studies should be followed.

Ionically conductive materials called hydrogels, inogels, or polyionic elastomers have been introduced as a new type of conductors that use charged ions rather than electrons to allow electrical signals [138]. In general, ion conductors have excellent stretchability, transparency, and biocompatibility [139]. For example, polyvinyl alcohol/hydroxypropyl cellulose/fiber hydrogel is fabricated with artificial nerves to deliver stable AC and tunable DC electrical signals in robot finger movements for complete recovery [32]. Conductive hydrogels are used as 3D printing materials and have the advantage of constructing complex shapes. The ability to efficiently collect strain and vibration signals has been proven through the fabrication of sensors with a complex structure [138]. Most of the research results are used as wearable sensors under *in vitro* conditions. So far, ionically conductive materials with excellent electrical conductivity and physical properties and at the same time excellent biocompatibility have not been introduced.

For electroceuticals, exploring conductive materials is a key strategy, but above all, the nerve conduit must be biocompatible, biodegradable, and biostable. Also, conductive materials and/or composites must be applicable to the manufacturing process of conduit shapes, surface treatment, and internal micropatterns for guiding axon sprout.

### 3.2. Fabrication of a 3D conduit structure

**Figure 4.**
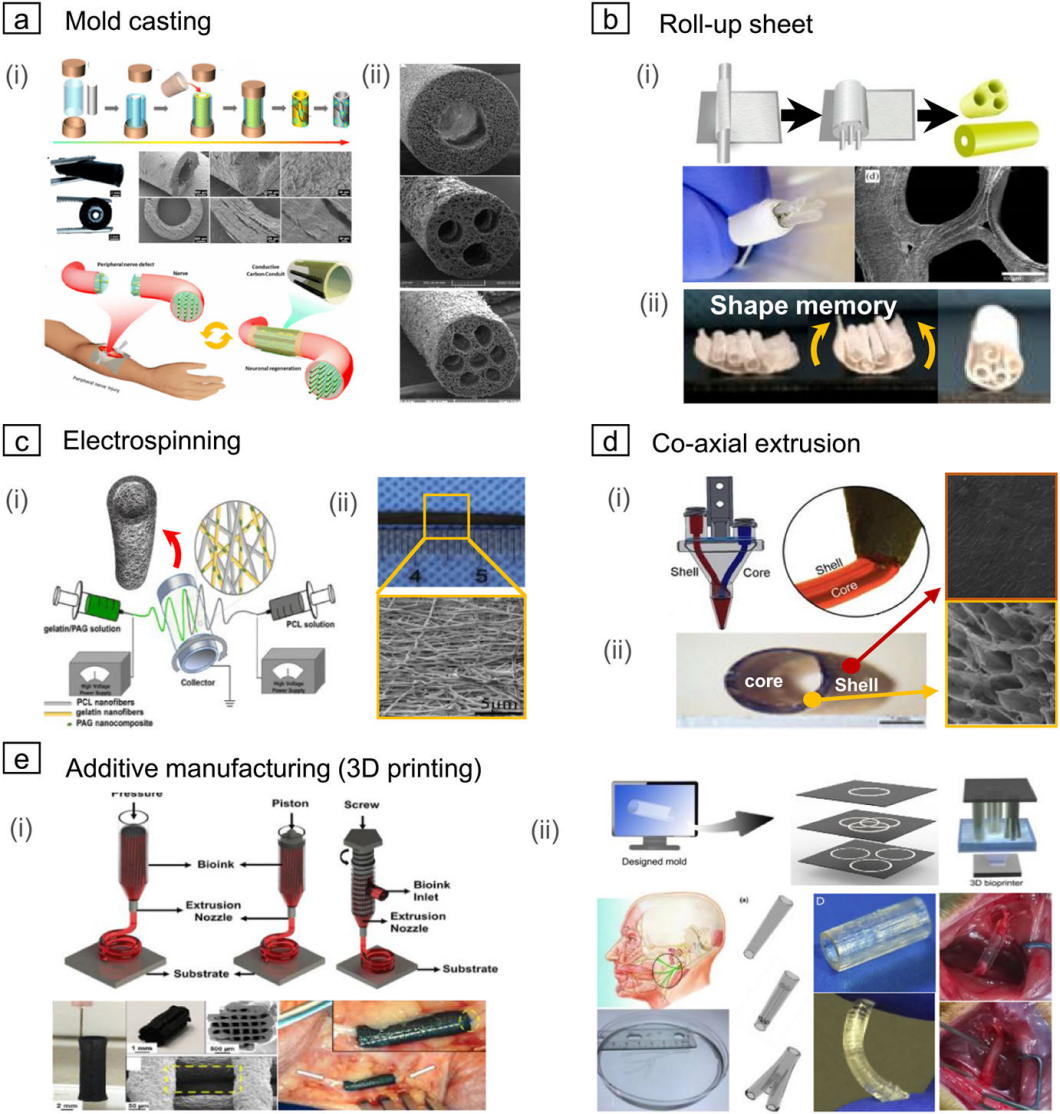
Various methods of constructing a nerve guidance conduit (NGC) with a cylindrical structure comprising single or multi-channel. (a) Mold casting; (i) representative illustration of the injection molding process and microstructure image of the manufactured conduit. Reprinted with permission from [140]. Copyright (2017) American Chemical Society. (ii) NGCs tube manufactured in various shapes according to the size and number of cores and location. Reprinted with permission from [141]. Copyright (2017) American Chemical Society. (b) Roll-up sheet; (i) schematic diagram of the rolled-up process using sheet and rod spacer (above) and manufactured conduit (below). [142] John Wiley & Sons. [original copyright notice]. (ii) Approach using electrospun shape memory nanofibers. The sheet keeps temporarily plane and then triggered by a physical temperature at 37 ° C to form a cylindrical conduit. Reprinted with permission from [143]. Copyright (2020) American Chemical Society. (c) Electrospinning; (i) illustration of dual electrospinning method. [142] John Wiley & Sons. [Original copyright notice]. Nano network conduits can be produced by jetting a polymer solution through a capillary nozzle with high voltage and depositing nanofibers in the collector. (ii) Photograph and microstructure of the electrospun NGCs. Reprinted from [144], Copyright (2019), with permission from Elsevier. (d) Co-axial extrusion; (i) schematic diagram of co-axial extrusion using a dual nozzle composed of inner and outer nozzles. Reproduced from [145], with permission from Springer Nature. (ii) The tube structure is produced by removing the core part of the extruded cylindrical filament. [146] Taylor & Francis Ltd. http://tandfonline.com. (e) Additive manufacturing (3D printing); (i) nozzle extrusion-based 3D plotting technique according to designed toolpath. [147] John Wiley & Sons. [Original copyright notice]. Reprinted with permission from [148]. Copyright (2015) American Chemical Society. (ii) Schematic diagram of the stereolithography based additive manufacturing using photocurable solution. Reprinted from [149], Copyright (2019), with permission from Elsevier. Stereolithography can provide customized therapy options for complex-shaped nerves defect. [150] John Wiley & Sons. [Original copyright notice]. Optical image of 3D printed NGCs, which has high flexibility. Reprinted from [151], Copyright (2018), with permission from Elsevier.

Fabrication methods of fine-scaled cylindrical shapes with conductive materials were a critical obstacle to facilitating the functionalized conduit in a clinical application. Mold casting can provide the simplest and easiest way to fabricate the hollow tube structure. More specifically, rods are placed in the core of the mold to create inner cavities. Then, injection of the prepared composite solutions into the rest of the casting mold serves hollow conduit structure, followed by demolding after complete solidification (figure 4(a) (i)). In addition, mold casting of a biodegradable polymer with carbon material can improve nerve conduction instead of non-conductive materials [106, 140]. The number of internal rods allows multi-channeled conduits as well as the single lumen, as shown in figure 4(a) (ii). The freeze-dried sponge-based conduit can even improve permeability by forming small pores on the cylinder wall [141]. The inner surface-to-volume ratio increased with more channels resulting in larger axon diameter and myelination thickness in the *in vivo* study.

However, the restriction of internal rods in various shapes and homogeneous channels should be overcome. It is also challenging to fabricate a microchannel because there is a risk of shape deformation or collapse during the demolding process via dissolving or pulling out. Therefore, optimization of the size, number, and position of internal rods will be a key feature to improve axon guidance as to future works.

A hollow structure can be created by rolling the sheet onto cylindrical rods. This strategy enables forming a conduit shape by pulling out the rod after wrapping it with a rectangular electrical conductive sheet. Figure 4(b)(i) shows that multi-channel conduits can be manufactured according to the size and number of rods for channel formation [142]. There is a limit to downsize to the required regenerated nerve. This handwork means atypical and may vary the guiding ability of axon sprouting depending on skill level. Here, there is an interesting study of the automatically roll-up method using shape-memory materials. When triggered by a core body temperature of 37 °C, it is restored to a tubular shape of a multichannel conduit (figure 4(b)(ii)). Angiogenesis and blood vessel formation could be promoted by forming a microchannel of the prepared sheet with nanofiber which provides more space for cell proliferation, migration, and neural tissue regeneration [143]. Although it is still difficult to construct a uniform structure with more fined channels, this has infinite potential with prepared nanopatterning for morphology cues.

Electrospinning is one of the most useful methods for producing nanofiber network sheets improving permeability during neuro-regeneration. The electrostatic field deforms the polymer droplets on the nozzle tip into a cone shape, and if the electrostatic force exceeds the surface tension, a charged jet is ejected. At this time, when a high voltage is applied to both the capillary nozzle tip and the substrate, the polymer nanofibers forward the ground plate of the counter collector, and a thin fiber network is deposited (figure 4(c)(i)). It generally has collected randomly nanofiber. Randomly nanopores only provide permeability, but not topographical cues. For this reason, various attempts have been trying to manufacture nanofibers with aligned directionality (figure 4(c)(ii)). Highly aligned vertical and horizontal, and randomly oriented nanofibers on the single matrix induce the directionality and proliferation of neural cells. Randomly oriented nanofibers allow increasing mechanical properties [152]. This method is an effective strategy that can provide directional guidance at the nanoscale. It can be applied in various ways, such as directly jetting using a composite solution containing conductive materials [126] or coating the collected sheet after electrospinning [95, 153]. Either way, well-fabricated nanofibrous substrates could offer superior potential on PNR by neuronal expansion and topographical guide cues.

Simultaneous extrusion of the two materials using a dual nozzle allows a hollow shell structure by selective removing the core part (figure 4(d)(i)). This cost-effective and rapid construction method is a useful technique to control the inner channel by controlling the materials’ feed rate. However, since a thin nozzle is placed inside the outer nozzle, if the outer shell’s space becomes narrow, the fluid may not flow due to the rheological limitation. As a result, it may be challenging to manufacture fine-scaled conduits. Figure 4(d) (ii) shows that a conduit has a diameter of about 2.5 mm, and the shell part is composed of a pore structure for permeability. The electrical conductivity, swelling, degradation rate, mechanical properties, and cell proliferation ability can be controlled by the composition of the shell material [146]. In general, co-axial extrusion has the advantage of easily controlling the thickness of the shell according to the extrusion feed rate of the core and shell, but it is difficult to construct multiple channels. Therefore, it is suitable for manufacturing artificial blood vessels as well as NGC that requires a single channel. It will be exponential effect doubled when collaborating with electrospinning or 3D printing techniques.

Previous descriptions of traditional methods for manufacturing conduits are suitable for producing standardized simple lumen shapes. Therefore, it is challenging to build the freeform shapes of conduits or handle a complicated structure. Additive manufacturing allows us to construct tailored complex geometry for individual nerve injuries.

Recently, for the nozzle-based 3D plotting method, the extruded filament is deposited through the nozzle to consolidate while moving along the preprogrammed tool path (figure 4(e)(i)). This technique makes it possible to print cell-laden filaments so that more bioactive structures can be constructed. The extrusion-based 3D printing method has a low resolution because the depends on the thickness of the extruded filament through the nozzle. Attempts have been fabricated to develop the resolution by increasing the extrudability with high temperatures to facilitate extrusion [154]. However, there are still challenges to mimicking nerves.

Alternatively, the studies related to the manufacture of scaffolds using a stereolithography-based 3D printing technique have been started. Stereolithography-based printing can fabricate a fine multi-lumen architecture because of its higher resolution than extrusion-based 3D printing. This advanced approach aims at customized treatment by accurately scanning and rapidly fabricating damaged nerve areas. It suggests that it can be built as human nerve-sized with complex structures (figure 4(e)(ii) above) [155]. There are several biodegradable, 3D printable composite solutions for artificial nerve fabrication. The synthetics of photocurable copolymers and water-soluble hydrogel composite have opened a new process paradigm for constructing microchannels in artificial nerves. This presents the possibility of constructing a physical space of under 450 *μ*m required for axon regeneration [156] unable to traditional fabrication methods. The conduit fabricated as shown in figure 4(e)(ii) below has sufficient flexibility and sufficient strength to resist compression modulus or sutures. A photo-initiator was added to produce a printable composite and reported unprecedented speed based on establishing a rapid continuous 3D direct light Pprocessing (DLP) printing platform [141]. Clinical application of 3D printed conduit upgrades fabrication technique and expands the scope of application of *in vitro* and *in vivo* platforms for personalized medical care for patients.

It has been reported to improve CNS regeneration via precisional medicine of biomimetic scaffolds such as the construction of 3D micro-architecture [157]. However, to our knowledge, there is no reports for manufacturing conductive conduits by stereolithography-based 3D printing by far. Therefore, this advanced manufacturing technique needs further study toward functional conduits for ES coupling, in terms of higher resolution and suitable properties for nerve bridges.

## 4. Integration of bioelectronic ES platform at the device level

Currently, it is limited to temporary and unsustainable stimulation through a wire-to-percutaneous electrode connection from an external power device. These percutaneous electrodes cannot be fully implanted, and the wires exposed to the outside of the skin may cause secondary infection after surgery, leading to a poor prognosis. Therefore, the needs of the fully fixation method are required for successful electroceuticals, which demands highly wireless power transfer. Here, we will introduce several wireless platforms, including energy harvesters from external power, actuators transmitting energy to tissue, and sensors for monitoring biosignals.

### 4.1. Energy harvester with wireless control

**Figure 5.**
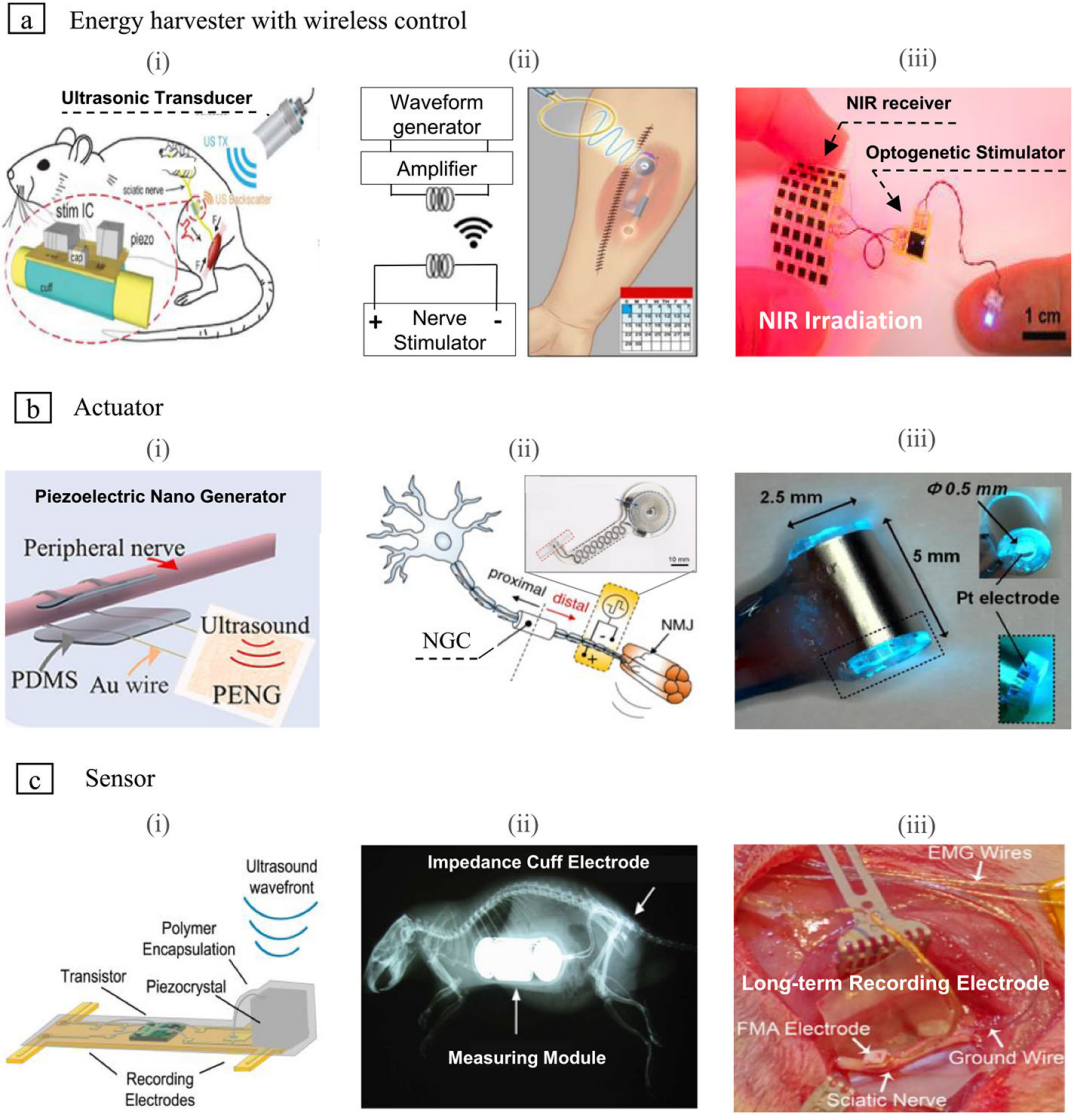
Integration between bioelectronics conduit and wireless platforms to efficient energy transmittance for improving neural regeneration. (a) Energy harvester with wireless control; (i) ultrasound-based wireless platform. © 2018 IEEE. Reprinted, with permission, from [158]. (ii) The induced current by radiofrequency (RF) signal from an external coil. Reproduced from [159], with permission from Springer Nature. (iii) Optogenetic wirelessly powered device by near-infrared (NIR) light. Reprinted from [160], Copyright (2021), with permission from Elsevier. (b) Actuator; (i) ultrasound-driven piezoelectric thin film nanogenerator. Reprinted from [161], Copyright (2021), with permission from Elsevier. (ii) Stretchable, bioresorbable electronic stimulator worked by induced current. Reproduced from [162], with permission from Springer Nature. (iii) Compact optical nerve cuff electrode for neural stimulation and monitoring. Reproduced from [163], with permission from Springer Nature. (c) Sensor; (i) nervous recording system with ultrasonic neural dust. Reprinted from [164], Copyright (2016), with permission from Elsevier. (ii) Long-term nerve impedance monitoring microsystem. © 2013 IEEE. Reprinted, with permission, from [165]. (iii) Multi-sites long-term recording electrodes. Reproduced from [166]. © IOP Publishing Ltd. All rights reserved.

Recent implantable bioelectronic device research has focused on miniaturized and wireless energy transportation to operate devices battery-free because of the limited capacity of it. To facilitate operating battery-free devices, there is an *in vivo* energy harvester (IVEHS) that accumulates the energy generated *in vivo* such as piezoelectric, triboelectricity, automatic wristwatch, biofuel, endocochlear potential, optical energy [167-171]. However, these have poor output, conversion efficiency, and poor durability [172]. Therefore, we need transmission from a stable external power supply to an efficient wireless platform of *in vivo* devices, and there are several strategies; ultrasonic (figure 5(a)(i)), induced current by radiofrequency (RF) (figure 5(a)(ii)), and optogenetics (figure 5(a) (iii)).

Since the wireless stimulation devices are fully implanted nearby the sciatic nerve, they must be fabricated minimalize and flexible. Figure 5(a) presents illustrations of each energy harvester. They have an external power source external to the body and wirelessly harvest power from a receiver placed inside the body. Through optimal matching between the transmitter and receiver, the triggered energy is transmitted via the receiver to the actuator contacted with the sciatic nerve. All types of harvesters turned out to be feasible to harvest enough power to activate nerve stimulation. There is a miniature wireless peripheral nerve stimulator (6.5 mm^3^) called stim dust operated by ultrasonic. They succeeded in converting harvested ultrasonic waves with 82% peak chip efficiency, indicating that they can be operated with low power [158]. In particular, in the case of figure 4(a)(ii), all substances are composed of bioabsorbable materials and absorbed from the body, and released out of the body after full recovery with nerve stimulation [159]. In addition, near-infrared (NIR) light is a highly efficient energy source that is optically driven and controlled since it has high transmittance in biological tissues (655–900 nm). The flexible system Integration (SI) product validation (PV) arrays that build up the device generate power when illuminated by NIR light. At this time, the stimulation is activated by transmitting a signal to the connected optogenetic stimulator. They show that remote control can be efficiently delivered to optogenetic devices wirelessly through skin tissue [160].

Compared to IVEHS, these devices need to generate external power, but they are stable wireless communication systems with high efficiency electrically and functionally. Although it has the challenge of further extending the working distance in the future, these are innovative technologies that can allow long stimulation periods enabling the complete implantation of devices.

### 4.2. Actuator

The harvested energy could be delivered to the *in vivo* tissue in various forms. Research on implantable electroceuticals related to signal transmission at the nervous system and nerve regeneration through electrical signals is actively studied. Figure 5(b)(i) presents the piezoelectric thin film nanogenerator connected to the cuff for sciatic nerve stimulation. In general, the potential for stimulus is quite weak with harvested energy from the movement of the body or organs. Here, a programmable ultrasonic-driven stimulator combined with a battery-free thin-film nanogenerator for peripheral nerves was introduced to increase electric power. The piezoelectric thin film nanogenerator using ultrasound as an external energy source successfully achieved direct ES of the sciatic nerve in mice [161].

In the Choi *et al* study, a flexible expansion electrode and a targeted peripheral nerve target device were constructed by connecting RF power harvester that receives electricity from an external induction coil (figure 5(b)(ii)). It is encapsulated with bioabsorbable dynamic covalent polyurethane, which has excellent mechanical elasticity for *in-vivo* movement and provides minimal expansion, enabling powerful operation without limiting its working lifetime. As for the electrical interface, the exposed Mo electrodes surrounding the nerve were connected to the PLGA tubular conduit to perform nerve stimulation efficiently. Energy harvester, electrode, and conduit are all absorbent materials, and after the initial ES treatment, they completely decomposed in the body. Unlike the previously known concept of proximal nerve stimulation for regeneration, this bio-device prove a beneficial effect applied to the distal part of the damaged nerve to improve muscle strength and function and increase peripheral blood to recover nerve damage [162].

Optogenetic stimulation of the peripheral nervous system is a novel approach to motor control, sensory transmission, and pain block. However, it needs to be careful about the temperature change during optical stimulation. When the temperature increases, partial heat causes block of nerve signals, which can lead to irreversible nerve damage [173]. Figure 5(b)(iii) shows an optical nerve device for both optogenetic stimulation and synchronous monitoring of peripheral nerve neural activity using a single cuff electrode. The role of optogenetics is to stimulate sensitive genes with specific wavelengths of light optically. The increasing temperature during optical stimulation led to fatalities, but this actuator was found not to affect nerve tissue damage during signal recording with changes only in the range of 24 °C–26 °C. The device was suitable for monitoring neural activity and optical stimulation in transgenic mice [163].

### 4.3. Sensor

A wireless system that integrates with external power supply and internal energy harvester is evolving into *in situ* sensors to monitor vital signs beyond the stage of tissue stimulation. This is to observe how to interpret and control the electrophysiological activity of the body to prove the therapeutic effect in the target tissue. In future work, it is expected to develop into a patient-customized medical device capable of nerve stimulation and sensing simultaneously.

Seo *et al* show the design of a miniature receiver device working by ultrasound system (figure 5(c) (i)). This neural sensor recorded the electromyogram (EMG) response elicited from the gastrocnemius muscle in rats. The data were recorded for 20 ms around the stimulation window through stimulation with 200 *μ*s duration and 6 s pulse. Monitoring could be continued indefinitely on the anesthesia, and there was no deterioration in the quality of the recordings even after continuous measurements for 30 min. Also, the difference between wireless and wired data was within ±0.4 mV, and the minimum signal detected by the sensor was about 0.25 mV. They proved that ultrasound effectively delivered energy to mm-scale devices through high-fidelity electromy-gram and electroneurogram signals [164].

Here, the Li group manufactured a neural impedance sensor for long-term monitoring of regeneration status for 42 d (figure 5(c)(ii)). Specifically, an implantable microsystem was inserted to observe the time-lapse variation of nerve impedance after wrapping the cuff electrode in an 8 mm injured sciatic nerve rat model. This system resulted in increasing myelin fiber density by offering long-term stimulation to accelerate nerve fiber growth as well as impedance evaluation to understanding as time goes by nerve regeneration. Continuous muscle stimulation can lead to better functional neural connections to reduce muscle atrophy and improve functional recovery [165].

The Vasudevan group further improved long-term monitoring by acquiring area mapping records with 16 sites records (figure 5(c) (iii)). They evaluated the changes in neural function through weekly *in vivo* impedance measurements and recordings. Although it was possible to achieve low-yield neural recordings of action potentials by floating microelectrode arrays in PNS, long-term recording performance was limited due to lead wire failure. After implantation, structural problems in which significant damage to the electrode and insulator, limiting continued reliability, but it is still expected to broadly expand the scope of application in the future by enabling evaluation and monitoring of the performance characteristics of neural arrays [166].

### 4.4. Optimize the therapeutic parameter

Despite the excellent neuro-regenerative effects of non-pharmacological electroceuticals, we still have not found a comprehensive and clinical therapeutic option. In the regeneration of damaged nerves, the degree of regenerative ability differs from the size and type of defect, location, age, and sex. Therefore, it is difficult to explain the set as ‘standard’ as the parameter of 1 h, 20 Hz, 0.1 ms for various conditions [85]. So far, evaluation is being performed under numerous healthy SD rats rather than clinical considerations. We need systematically strict investigation with more detailed conditions through multiple simple models. We may need the help of biocomputational tools for optimizing ES parameters and device design using artificial intelligence and machine learning in the future. But, this is a challenging issue because it needs numerous training samples are required for the utilization of deep learning applications. One of the approaches is to collect training data from computer simulation experiments. Another one might be meta-learning which learns to quickly adapt to a new environment with just a few samples [174]. Ultimately, optimizing parameters is a ‘key factor’ for boosting the therapeutic effect and applying clinical application.

## 5. Challenges and future works

Despite the remarkable potential of conductive NGCs to deliver ES for nerve regeneration, there is few optimal guideline of electroceuticals. As shown in table 1, the electrical properties (e.g. conductivity, impedance, resistance) employed in each research vary extensively. The biological reaction at nerveconduit interface might also increase the conductive variation, such as impedance and biofluid acumination. In addition, the evaluation of the electroreaction to the disrupted nerve being connected to the NGCs is not standardized. Furthermore, it should be recognized that the surface of NGCs is exposed to the bio-fluid directly, which does not fully deliver charges by causing electrical leakage in *in vivo* environment. Actual axons are encapsulated by an insulator called the myelin sheath, which increase neural activity of axon and prevent the leakage of electric signal. However, most conductive conduits are designed without this precise structure, which may cause a decrease in the efficiency of ES. In addition, the technical limitations of fabricating inner channels in a micro-scale allows only single-lumen type structures as nerve bridges, rather than multichannel structure. Researches on optimal pore size and channel structure through material combination and fabrication process are also important factors to increase mass transportation of nutrients and oxygen which are essentials for nerve regeneration. In a long-term implantation to mimic severe nerve injuries, the large nerve gap (>10 mm) requires sustainable mechanical and electrical properties during the designed degradation lifetime. The conductive and bioresorbable materials, including conductive hydrogels within the hollow NGCs should be carefully evaluated to prove the complete biosafety and biocompatibility, according to well-established protocol.

From the clinical perspective, the bioelectronic platform should be biocompatible, bioresorbable, miniaturized, and with ease of implantation under minimal invasive procedures. Different from other bioelectronic device designed for brain or spine, strong mechanical sustainability with stretchable property is crucial for peripheral nerve system, owning to its nature of high degree of mobility and pressure loading. Considering about the possibility of incomplete nerve regeneration or the formation of neuroma-in-continuity at the critical nerve gap, the detection of nerve action potential via implantable bioelectronic device can bring decisive information for the progress of nerve regeneration. In order to further investigate the underlying mechanisms of ES on PNR, several advanced technology can be expect to explore the full spectrum of peripheral nerve system, such as (a) single cell RNA sequencing for better understanding of cell responses, cell-cell interactions with unprecedented spatial and temporal resolutions; (b) biocomputational tools such as artificial intelligence and machine learning to help optimize the therapeutic parameters and device design. Furthermore, the versatile adjustment of the dosage of electroceuticals according to real-time nerve sensing will open a new era of precisional medicine as theranostic bioelectronic platform for electroceuticals in the future.

## 6. Conclusions

Both bio-engineered nerve conduit or electroceuticals has shown beneficial evidences in PNR for 20 years. Recent advances on biomaterials empower the modern bioresorbable and conductive nerve conduit, which enables to deliver versatile therapeutic electroceuticals. The biofabrication to integrate conductive nerve conduits with ES platform can offer versatile stimulation dosage, depending on the injury type, timing and progress of nerve regeneration. The modern bioelectronic platform for electroceuticals integrates the cutting-edge technology of tissue engineering and biofabrication to develop the future theranostic bioelectronic devices for regenerative precision medicine.

## Figures and Tables

**Table 1.** Summarized parameters of current studies with engineering conductive conduit and ES for PNR.

Materials	Conduit fabrication	Electrical properties	ES condition	Cell	Animal	References
PPy/chitosan	Injection molding technique	(1.5 ± 0.2) × 10^−2^ S cm^−1^	3 V of 20 Hz, 0.1 ms for 1 h	No data	Male SD rat sciatic 200–220 g, 15 mm defect	[90]
PPy/PLCL	Electrospun	6.72–2.41 × 10^−5^ S cm^−1^	100 mV for 1 h/1, 3, 5 d	PC12, BDNF, GDNF, NT-3 mRNA	SD rat sciatic 15 mm defect	[95]
PPy/PLGA	Electrospun	(7.4 ± 3.2) × 10^3^–(9.0 ± 6.0) × 10^4^ Ω sq^−1^	10, 100 m V cm^−1^ for 2 h	PC12	No data	[96]
PPy/PCLF	Injection molding technique	2–25 kΩ	10 *μ*A of 20 Hz 1 h d^−1^	PC12	No data	[97]
PPy/SF	Electrospun	114.46–3.98 × 10^−3^ ms mm^−1^	3 V of 20 Hz, 0.1 ms for 1 h	SCs	SD rat sciatic 180–220 g, 10 mm defect	[98]
PPy/PDLLA	Roll-up sheet, emulsion technique	5.65–15.56 ms cm^−1^	100 mV for 2 h	PC12	SD rat sciatic 150–200 g, 10 mm defect	[99]
PPy/BC/N-CNTs	Sheet with elecrospun conduit	1.51 ± 0.05 S cm^−1^	Self-powered stim. Similar to 50 mV mm^−1^	DRGs	Female SD rat sciatic 220 g, 15 mm defect	[100]
PPy/PECA, PPy/PCL	Airbrushing, pressing, roll-up	19–32 S cm^−1^ 10^3^–10^4^ Ω sq^−1^	100 *μ*A for 2 h	PC12	Male SD rat sciatic 350 g, 10 mm defect	[101]
PPy/SA/CMCS	Nerve conduit filling material	2.41 × 10^−5^–8.03 × 10^−3^ S cm^−1^	100 mV for 2 h d^−1^	PC12, RSC96, BMMSC	SD rat sciatic 150 g, 10 mm defect	[102]
PANI/PCL/gelatin	Electrospun	0.02 × 10^−6^ S	1.5 V for 15, 30, 60 min during 1, 3, 5 d	NSC	No data	[103]
PANI/HEC/SPI	Injection molding technique	1.7 S m^−1^ 20.6 kΩ sq^−1^	3 V for 1 h 7 times during 2 d	SCs, BDNF, MBPs	SD rat sciatic, 180–220 g, 10 mm defect	[89]
CNT/Sericin	Injection molding technique	(3.73 ± 0.51) × 10^−5^–(3.90 ± 0.26) × 10^−4^ S cm^−1^	3 V of 20 Hz, 0.1 ms for 1 h	RSC96	Male SD rat sciatic, 200–300 g, 10 mm defect	[104]
SWCNT/SF	Roll-up sheet manufactured by electrospun	Approx. 2.1 × 10^−3^ S m^−1^	1 V of 2 Hz, 0.2 ms	U373-MG	Male SD rat sciatic, 200–250 g, 10 mm defect	[105]
rGO/GelMA	Injection molding technique	1.1 ± 0.1–8.7 ± 1.6 mS cm^−1^ 10 ± 1–193 ± 13 kΩ	Stim. X Electromyogram (EMG) signal 1.5 V	PC12	Male SD rat sciatic, 180–200 g, 10 mm defect	[106]
rGO/ApF/PLCL	Electrospun	1.96 × 10^−3^–4.05 × 10^−2^ S m^−1^	DC 100 mV cm^−1^ for 1 h d^−1^	SCs PC12	Male SD rat sciatic, 200–250 g, 10 mm defect	[107]
CNT/PPDO	Electrospun	1.73 × 10^−10^, 1.44–3.52 × 10^−6^ S cm^−1^	50 mV mm^−1^ for 1 h d^−1^	hADMSCs	No data	[108]
MXene/PCL	Electrospun	2.91 × 10^−2^ S cm^−1^	Self-originated ES	RSCs	Male SD rat sciatic, 150–200 g, 15 mm defect	[109]
rGO/GelMA/BDNF/*Morpho menelaus*	Roll-up sheet	Approx. 0.5–1.0 S cm^−1^	Stim. X.	PC12, NSC	Male SD rat sciatic, 180–250 g, 10 mm defect	[110]
ZnO/PCL	Electrospun	40–120 mV	Piezoelectric	RSCs	8 weeks old SD rat sciatic, 10 mm defect	[111]

PPy; polypyrrole, PDLLA; poly(d, l-lactic acid), PCLF; polycaprolactone fumarate, SF; silk fibroin, PLCL; poly(l-lactic acid-co-*ε*-caprolactone) BDNF; brain-derived neurotrophic factor, GDNF; glial cell derived neurotrophic factor, NT-3; neurotrophin-3, PLGA; poly(lactic-co-glycolic acid), BC; bacteria cellulose, DRGs; dosal root ganglion cell, N-CNTs; nitrogen-doped CNTs, SWCNT; single wall CNTs, PECA; poly(ethyl cyanoacrylate), PCL; poly(*ε*-caprolactone), SA; sodium alginate, CMCS; carboxymethyl chitosan, PANI; polyaniline, HEC; hydroxyethyl cellulose, SPI; soy protein isolate, rGO; reduced graphene oxide, GelMA; gelatin-methacrylate, ApF; Antheraea pernyl silk fibroin, PC12; rat phaeochromocytoma cell line, SCs; Schwann cell, RSC96; Schwann cell, BMMSC; bone marrow mesenchymal stem cell, MBPs; myelin basic protein, NSC; nerve stem cell, U373-MG; human glioblastoma-astrocytoma, SD rat; Sprague–Dawley rat, PPDO; poly(p-dioxanone), hADMSCs; human adipose-derived mesenchymal stem cells, MXene; Ti_3_C_2_T_*X*_, RSCs; Rat Schwann cells.

## Data Availability

All data that support the findings of this study are included within the article (and any supplementary files).
